# Recovering from Hallucinations: A Qualitative Study of Coping with Voices Hearing of People with Schizophrenia in Hong Kong

**DOI:** 10.1100/2012/232619

**Published:** 2012-12-11

**Authors:** Petrus Ng, Ricky W. K. Chun, Angela Tsun

**Affiliations:** Department of Social Work, Hong Kong Baptist University, Hong Kong

## Abstract

Auditory hallucination is a positive symptom of schizophrenia and has significant impacts on the lives of individuals. People with auditory hallucination require considerable assistance from mental health professionals. Apart from medications, they may apply different lay methods to cope with their voice hearing. Results from qualitative interviews showed that people with schizophrenia in the Chinese sociocultural context of Hong Kong were coping with auditory hallucination in different ways, including (a) changing social contacts, (b) manipulating the voices, and (c) changing perception and meaning towards the voices. Implications for recovery from psychiatric illness of individuals with auditory hallucinations are discussed.

## 1. Introduction

Schizophrenia is a major mental illness in contemporary society which affects about 1% of the world population [1]. People in an acute episode of schizophrenia are characterized by the positive symptom of auditory hallucinations or voice hearing. The voices, particularly those with negative and critical connotations, would directly or indirectly affect their emotional, economic, occupational, and social functioning. In Hong Kong, while psychiatric medication is the primary treatment, there are no specialized services for Chinese patients with the problem of hearing voices. With the influence of traditional Chinese cultural beliefs, they may worship gods, drink “amulet tea,” or adopt methods associated with folk religions or customary lay practices in trying to copepassively or actively with the voices. As there has been no previous study on the hearing of voices by Chinese people with schizophrenia, this paper examines their coping strategies with an emphasis on how they adopt lay practices within the sociocultural context of Hong Kong.

## 2. Schizophrenia in the Chinese Sociocultural Context

Schizophrenia is a group of disorders which severely disrupts the memory, visual and auditory perceptions, problem-solving, social, and cognitive abilities of the persons affected [2, 3]. In a study of people with schizophrenia in Beijing, China, it was found that the general lifetime prevalence of the illness was 0.49% and 0.44% for men and 0.55% for women [4]. There is no comprehensive study on the lifetime prevalence of schizophrenia in Hong Kong. However, a 15-year outcome study of 100 people with schizophrenia found that about 10% had committed suicide though about 50% had experienced improved symptomatic outcomes [5, 6]. There has been very little research on voice hearing of people with schizophrenia in different environments and cultures. In Chinese culture, the traditional beliefs of Chinese medicine, which emphasize the balance of yin and yang and proper proportions of five elements, including wood, fire, earth, metal, and water, have considerable influence on people's perception of health and mental health [7]. Imbalance between yin and yang and among the five elements will result in illness or psychopathology [8, 9]. There is not a distinct mental disorder called schizophrenia according to traditional Chinese medicine, and mental illness is broadly divided into two categories, namely, “Kuang”— psychosis with excitation and “dian”—psychosis without excitation or epilepsy [10, 11]. 

In addition to traditional Chinese medicine beliefs, spirit possession and retribution for sinful deeds are other commonly held folk beliefs related to the etiology and treatment of madness by lay persons in Chinese societies, such as Hong Kong [7, 12], Taiwan [13], and Singapore [14]. Chinese mental patients often attribute their problems to possession, to a charm cast on them, or to having stepped on a spirit or a “dirty thing” accidentally. Chinese people also have the folk religious belief of retribution for sin in the form of a bad spirit that would invade the body and inflict madness when misdeeds and human transgression, such as family conflict, occurred [7]. 

Confucian thought is another traditional belief that shapes Chinese people's experience of mental health problems. Mental illness was regarded as a punishment for violating the Confucian norms governing interpersonal relations, especially filial piety [15], and ancestors could become malevolent spirits to haunt their descendants who had violated Confucian teachings [7]. 

## 3. Coping with Auditory Hallucinations

Although individuals with schizophrenia were often thought to be passive victims of auditory hallucinations [16], researches have identified the difference between the coping strategies of the “copers” and the “non-copers” [17–19]. The “copers” could manage well and feel themselves to be stronger than the auditory hallucinations. They could experience the voices as positive and could experience fewer comments from them. The “non-copers,” on the other hand, experienced the voices as negative and aggressive. The “copers” were found to engage more often in selective listening, such as listening only to the positive voices, and were more able to ignore them, while the “non-copers” more often tried to utilize distraction techniques. In general, changes in social contacts and manipulating the level of sensory stimulation were the two basic coping strategies identified for the “copers” [19–21].

Changes in social contacts included increased social contacts and engaging in social conversations. There were two processes underlying this coping strategy. First, engaging in a conversation with others acts as a form of distraction that might help persons experiencing hallucinations to keep their minds focused and their attention distracted from the contents thereof [21, 22]. Second, engaging in conversation through verbalization on hallucinations could serve as a protective factor for psychological dysfunctioning [23]. As auditory hallucinations are often accompanied by subvocalization [24], concurrent verbalization helps suppress the sub-vocal speech and to reduce the severity of hallucinations [25]. Increasing social contacts therefore served a dual purpose, that is, (a) distracting oneself from the content of distressing hallucinations and (b) disrupting the subvocal activity concomitant with hearing voices. Although an increase in social contacts was frequently reported, some patients might actually withdraw from social contacts in order to cope with their hallucinations [19]. This was particularly true for patients with relatives highly expressive of their emotions when contacts with the latter were sources of their stress. 

Manipulating the levels of sensory stimulation was accomplished by minimizing sensory input, such as by closing the eyes or putting plugs in the ears, or by increasing other sensory stimulations [20, 21], such as listening to loud, stimulating music [19, 20, 26, 27]. Decreases in internal auditory hallucinations, such as threatening and critical comments, with increased external auditory stimulations through the use of a portable cassette player, have proven to be a popular form of coping strategy for people who hear voices [25, 28, 29]. The meaning of the stimuli, such as meaningful sound, music, and interesting excerpts of speech, was important in helping to reduce the frequency of auditory hallucinations [26]. 

In addition to the change in social contacts and manipulating the levels of sensory stimulation, developing a meaning of the voices was another significant means to cope with voice hearing. Apart from reducing ambiguities, developing a meaning of the voices would reduce likelihood of error in the attribution of the source of one's perceptions. Thus, listening to music with a meaningful pattern of sounds, preferably at a high volume, would help to reduce hallucinatory activity and the feeling of powerlessness of patients [29, 30].

Perhaps the most important foundation of these coping strategies is based on the conceptualization of the individuals as active goal-directed agents who can influence the course of their voice-hearing problem. Thus, in focusing our view on the strength rather than on the problem and its symptomatology of the individuals, this study attempts to get a more complete picture of the course and coping strategies of voice-hearing problems of Chinese people in Hong Kong. 

## 4. Method

### 4.1. Participants and Procedure

The objective of this paper was to examine the coping strategies on auditory hallucinations, of Chinese people with schizophrenia in Hong Kong. As the coping with the problem of hearing voices is subject to the hearers' own interpretations, a qualitative research method therefore was adopted to facilitate the participants' ability to describe the phenomena of the voices in fine detail and in their own terms. The qualitative approach was used to gain a deep understanding of the complex subjective experiences people afflicted with the problems [31]. With respect to sample size, McCracken suggested that eight respondents would be sufficient for many qualitative projects [32] while Creswell thought that ten subjects in a study represented a reasonable size [33]. In view of the qualitative and exploratory nature of investigating the subjective experience of the voice hearers, purposive sampling was employed to select participants who met the criteria for the study. The inclusion criteria for the participants included the following: (a) that they had received a definite diagnosis of schizophrenia, (b) that they were 18 years of age or above, (c) that they had had the illness for more than 6 months, (d) that they has been clinically stable for at least 1 month prior to assessment (clinical stability is operationally defined as absence of exacerbation of illness requiring an increase in drug dosage by 50% or more), and (e) that they had reported the presence of auditory hallucinations. The exclusion criteria included (a) patients with diagnosis of organic brain syndromes, substance abuse, subnormal intelligence, and (b) those having difficulty in verbal communication. 

 The participants were recruited through nongovernmental organizations (NGOs) providing services for people with mental illness in Hong Kong. Invitation letters and a brief research proposal were sent to the directors of five NGOs for ethics review and approval. Finally, two NGOs gave positive responses to the invitations. With the assistance of the social workers of the two NGOs, individuals who met the inclusion criteria were invited for interviews with their consent. Altogether 20 participants from these two NGOs were recruited for the study. 

### 4.2. Data Collection

This study was based on in-depth interviews with 20 schizophrenia patients with auditory hallucinations. Before interviewing the participants, pilot interviews with reference to “Interview with a Person who Hears Voices” [34] were conducted with two Chinese adults with schizophrenia. The results of these pilot interviews were of help in developing a semistructured interview guide which included 4 areas of inquiry, including (a) general characteristics of the voice hearers, (b) content of the voices and attitudes about the voices, (c) impacts of the voices on the hearers, and (d) coping strategies with voice hearing. The draft interview guide was developed with reference to pre-study interviews with social workers working with people with voice hearing and clients with voice-hearing problems in 2 NGOs and was sent to social workers of the NGOs for comments. The interviews took place in the interview rooms of the two NGOs on a face-to-face basis. To prevent overly long interviews and to avoid overtaxing the subjects, the in-depth interviews were broken into two to three shorter sessions, each lasting normally for 45 minutes to an hour.

### 4.3. Data Analysis

With the consent of the participants, the interviews were digitally recorded. With the assistance from a student research helper, a verbatim transcript of each interview was prepared in Chinese so as to faithfully record the original form of expression of the participants and allow cross-referencing during the process of analysis. Observations, reflections and remarks concerning the codes were noted, sorted, and sifted in order to identify similar points, phrases, patterns, themes, and relationships of the voices and voice hearers.

The objective of qualitative analysis is to determine the categories, relationships, and assumptions that inform the participant's experience on voice hearing. To maintain the reliability and validity of the data, all interviews were digitally recorded with the consent of the participants. The interviews were first replayed, and verbatim transcript of each interview was prepared in Chinese with the assistance of two social work students. The reason for preparing a Chinese version of the transcripts was to faithfully record the original form of expression of the participants in the interviews and allow cross-referencing during the process of analysis.

The verbatim responses of 20 participants were then organized. First, with the help of computer, a data input table was constructed, and codes were affixed to the information from the transcripts. Second, observations, reflections and remarks were noted and cross-referenced with sociodemographic characteristics, such as length of the illness, social relationships, and internal and external resources of the participants. Third, the materials from the data table were sorted and sifted in order to identify similar points, phrases, patterns, themes, relationships, sequences, or differences between different groups of data. Finally, common themes or patterns, commonalities, and differences in the participants' experiences in voice hearing were identified. 

## 5. Results

### 5.1. Profiles of the Participants

There were 12 men and 8 women, ranging in age from 22 to 55, who participated in the study. The mean age of the males was 44.1 years, and that of the females was 27 years. All the participants were living in half-way houses and were receiving comprehensive social security assistance (CSSA). Most participants were unmarried. Five were working in sheltered workshops, and five in day activity centres; 10 were unemployed (see Table 1). All participants were receiving psychiatric follow-up treatment and were currently on psychiatric medications. Nine of them had had the first onset of illness before they were 17 years old. Most had been ill for over 20 years. All had a history of hospitalization or admission to mental hospitals.

### 5.2. Coping with Auditory Hallucinations

While the impacts of auditory hallucinations might affect different people in different degrees, there are variations of the adoption of strategies to cope with the voices of the hearers. In this study, participants had expressed different coping experiences in their adjustment toward hearing voices.

### 5.3. Changing Social Contacts through Ignoring and Justifying Voice Hearing with Lay Practices

Many participants were trying to ignore the strange new social experience particular at the early stage of voice hearing. The voices were so frightening, and the experience was so unfamiliar that they might retreat to their nut shell so as to reduce confusion. Ten participants of this study were admitted to mental hospitals as a result of the confusion caused by auditory hallucinations. They described the onset of the auditory hallucinations as being quite sudden, startling, and anxiety-provoking. Participant Alfred, for example, was admitted to a mental hospital as he was frightened by the voices:

*I was so frightened by the voices in my head. A doctor in the hospital explained to me that the voices I heard were auditory hallucinations… While in hospital and under medication, my voice hearing decreased… I believed that my brain was sick and that the voices were being released by my sick brain.*

Some participants were accompanied by family to seek medical treatment as Participant Catherine related:

*I was frightened by the voices and lost my temper with others. I tried to ignore the voices but could not do so… My mother got me a medical appointment and then I was admitted to a mental hospital. *

Apart from medical treatment, some participants and their family members believed that their voice hearing was related to folk religion and thought that worshipping gods in a temple could help cure the illness. Participant David remarked:

*My mother and I believed that the voices came from ghosts and made me confused. I went to meet a medium master who told me that the voices were induced by evil spirits. He suggested that I worshipped Chinese gods on the Mainland and took the amulet tea prescribed by the temples… The voices went away for a short time but returned after a month. I was then brought by my brother to seek medical treatment… I have been taking drugs for over ten years. However, I still feel confused about the causes of my voice-hearing in terms of spirits and mental illness.*

Many participants had tried different methods to deal with their voices but in vain. Take participant Gilbert, for example, who had tried a variety of strategies to cope with the voice hearing, but the strategies were not effective:

*I ignored the voices but in vain. I committed crimes after following the instruction of the voices. Then I was admitted to the hospital. I took medication in the hospital but I still listened to voices. I also tried to worship a Taoist god and the Buddha as well as my deceased paternal grandmother in different temples. However, all my efforts ended in failure.*

Many people with voice hearing experienced fear and confusion at the beginning of the illness. Their emotion, interwoven with folk beliefs, affected their help-seeking behaviour. Despite their efforts to deal with the problems, such as worshipping gods or drinking amulet tea, many people with auditory hallucinations ended up in hospital treatment. 

### 5.4. Manipulating and Regulating the Voices

Once the patients were able to deal with the basic problem of fear of voice hearing because it prompted concern as to whether they could feel safe and secure, they began to lock horns with the task of trying to find ways to cope with the voices. Learning to regulate the voices appeared to be a useful coping strategy for many participants. However, this could last for months or years for some hearers. In this study, some participants developed effective coping strategies, such as regulating the voices, or entering into willingness to dialogue with them by listening to them selectively. Many participants were able to reorganize their voices and became willing to comply with psychiatric treatment. In this study, all participants had received psychiatric treatment for several years. Compliance to drug treatment was conducive to good coping with voice hearing, as Participant Alfred observed:

*Before psychiatric treatment, I always looked for a source of my voices, such as people nearby, staff of a telephone network company, computers or other “spying devices”, and I never thought of mental illness… I had more insight into the voice hearing and learnt after the treatment that the voices were due to my mental illness. I considered drug treatment the most effective way to stop the voices and I complied with drug treatment.*

As a result of their side effects, psychiatric medications were often the last resort for dealing with voice hearing after the participants had struggled with the voices for some years. A number of the participants had tried their own folk or layman strategies to cope with auditory hallucinations themselves. The common layman strategies of the participants are shown in Table 2. Sometimes they found the strategies effective and sometimes they did not, as Participant Alfred reiterated, stating that he found his techniques of coping with the voice ineffective:

*I have tried to ignore the voices, ask the voices to go away, set the boundaries with the voices, and use distraction, such as engaging myself in other activities. They don't seem to work well… I give two scores for jotting down the content of the voices and covering my head with a blanket, and I give 5 as the highest score for paying attention to what the voices said to me.*

Although the coping strategies might not work for one person, there are no “supposed to's” in the process that lead us to assume that the same would happen to other persons. Participant Bobo said:

*I ignored the voices or tried not to listen to them but it was just one time out of two that I found these strategies useful. I then tried to scold the voices in my head, and sang to them about four times a week, and this turned out to be the most effective method… I could not reject the voices but gradually accepted their existence though I did not like them.*

There were also other strategies, such as going to bed, reading books, listening to songs, going to the library, trying to relax, and talking with family members; however, they were found not to be very effective in coping with the voices most of the time, as Participant David noted:

*I ignored the voices when I was not interested in them… I found it more effective if I sometimes argued with the voices. I was happy when I won through my sound argument and the voices would stop at times… I also set boundaries with the voices so that the voice identities only giggled to themselves instead of speaking to me… I consider my active reaction to the voices was the most effective way to deal with them.*

In general, people with voice hearing in the organizing phase have developed different layman strategies to cope with the voices, These strategies, effective or not, could be divided into two categories, namely, (a) passive and avoidance strategies and (b) assertive and interactive ones. As a strategy might be effective for one person and not for another, it is important for the voice hearers to get to know more about their own strategies and expand their repertoire thereof over time.

### 5.5. Changing Perception and Meaning toward the Voices

Another important way that participants influenced the course of their voice hearing was through a shift in perception and meaning which could lead to alternation of their help seeking behaviors. An important aspect of this shift appeared to be the development of a balance or compromise between the voices and themselves. With the new meaning towards the voices, they might consider the voices as a part of themselves and of their lives and could feel more in control of the voices. There is a growing ability to accept the voices as inevitable and to trust the professionals and receive help and treatment from them, as made clear by Participant Alfred:

*I did not share my voice hearing experience with others as I believed that others did not know about my (mental) illness and would look down on me. I would only talk about my voice hearing to those understood my illness… Now, I can take the initiative to tell my psychiatrist about my voices though I would not share with my friends who are ignorant about my illness.*

There is also a growing awareness of the significance of the impacts of the voices and of finding ways to solve problems in daily life that are linked to voice hearing. They had the confidence to talk about their voices, particularly with professionals, such as psychiatrists and social workers. Participant Catherine put it this way:

*I felt more relaxed and happy after sharing my voices with the psychiatrist and social worker… I got more insight about my voices from them and I was more confident of managing the voices… The social worker explained to me that the voices were hallucinations and encouraged me to communicate with my family members, friends, and advised me to find something to occupy myself during the day. I found such support and supportive communication to be effective in managing my problems.*

With the insight, Catherine began to accept her voices and to see professionals as persons she could trust and from whom she could receive proper treatment. As the acceptance of the voices gave the hearers a certain measure of autonomy in reacting to and dealing with them, they were better able to evaluate their relationships with the voices and to solve problems related to them on a reality basis.

## 6. Discussion

In Hong Kong, schizophrenia has constituted a long-recognized mental disorder and accounted for the largest number of persons receiving psychiatric recovery services [35, 36]. As auditory hallucination is a major psychotic symptom of schizophrenia, it has salient impacts on the lives of the sufferers. The experiences shared by the participants in this study highlighted the day-to-day struggles of persons with auditory hallucinations in the Chinese cultural context of Hong Kong. Results from this study showed that not only did the participants struggle with the voices, but they also had to bear the negative consequences in isolation. In general, the voice hearers were psychologically burdened, prone to odd behavior, financially disadvantaged as a result of poor employability, and socially isolated from family members and friends. They suffered great emotional strain with limited social support.

Hearing voices that no one else can hear can be disturbing and frightening for the hearers and for those around them. Family members and friends may have difficulty in accepting that the persons they care about are experiencing voice-hearing problems. No one really knew why people had auditory hallucinations. However, the participants in this study made use of some effective lay coping strategies, including (a) changing social contacts through ignoring and justifying the voices, (b) manipulating and regulating the voices, and (c) changing perception and meaning towards the voices. Moreover, the effectiveness of the strategies was related to the respective individuals' characteristics and might vary according to different phases of the problems. The results of this study have provided an initial understanding of coping strategies of people with auditory hallucinations in Hong Kong.

### 6.1. Limitations of the Study

Each research method has its limitations and strengths. The great strength of the qualitative method used in this study is the rich abundance of data on the subjective experiences of the participants obtained from in-depth interviews. However, in spite of the potential advantages of qualitative methods, there are situations that could undermine the validity of the study. Most pertinent in this regard is the fact that its qualitative nature meant that only 20 participants were interviewed. A small sample is unlikely to be representative of the population of the voice hearers in Hong Kong. Thus, the findings of this study cannot provide the basis for conclusions supported by statistical analysis, as with quantitative methods. 

Because of the impact of schizophrenia, the side-effects of drugs and perceived secrets of the voices, some participants might not be totally open to disclosing their hallucinations. Understandably, it was not easy for some of them to disclose freely the private contents of their hallucinations in the interviews. Owing to the nature of their illness, the answers given by some voice hearers were short and not necessarily relevant to the study. Again, the researcher had to rely on some follow-up questions in the interviews. It is therefore possible that some participants might not have been able to tell their story in their own terms, and this might well have affected the effectiveness of the study.

## 7. Implications for Practice

For many people with auditory hallucinations, coping with the voices could be a nightmare. In this study, we found that many Chinese people with schizophrenia were coping with the voices by adopting layman strategies. The findings from this study have generated insight and directions for social services to help people with auditory hallucinations. These include (a) developing a respectful attitude toward voice-hearing experiences, (b) developing culturally sensitive interventions for voice hearing, (c) formulating a specialized treatment programme for voice hearers, (d) enhancing the training for mental health professionals working with voice hearers, and (e) providing family education for family members of the voice hearers.

### 7.1. A Respectful Attitude toward Voice Hearing Experiences

In this study, we found that the voices were, to a significant extent, meaningful to the hearers, a fact that might have been disregarded by the professionals working with them, such as psychiatrists and social workers. The professionals should have a respectful attitude toward the hearers' voice hearing experiences. The voices themselves might contain a considerable fund of information about the individuals' unresolved feelings and conflicts. More in-depth understanding about the voice experience would help achieve insight into hearers' problems and direction of intervention from their perspectives. Professionals should not simply use a preconceived frame of reference or medical jargon to negate the hearers' voice experiences. In dealing with the subjective voice experiences of the hearers, they have to be patient so as to develop a relationship of trust with the hearers. With the establishment of such rapport, they become able to encourage the hearers to talk freely about their experiences in therapy sessions. It was found that even in such research interviews, a respectful attitude toward their voice experiences proved helpful to hearers in obtaining some insight into their voice experiences. 

### 7.2. Culturally Sensitive Interventions

 Auditory hallucinations are not totally meaningless, and they cannot be understood in a social vacuum. Understanding the social and cultural contexts is important for constructing, defining, and interpreting the reality that people with mental illness perceive and how they interpret their auditory hallucinations [37]. To be effective in helping people with voice hearing in coping with their auditory hallucinations, the interventions must build up a comprehensive understanding of community sanctions and social values as well as the life circumstances of the hearers. Thus, mental health professionals have to equip themselves with sensitivity toward indigenous heritages and social practices so as to prevent stereotypical or “supposed to” assessments and treatment. An indigenous culturally sensitive approach is needed for better therapeutic relationships and understanding of the hearers [38].

### 7.3. Specialized Programmes for Voice Hearers

Although psychiatric medication is still the primary treatment for auditory hallucinations, voice-hearing problem of many hearers persists. Helping the hearers to accept the voices on a basis of coexistence is a helpful way to defuse their unwanted internal sources of distress. In Hong Kong there is no specialized treatment programme to help voice hearers to cope with auditory hallucinations [39–41]. However, as there are more people with schizophrenia living in the community, there is a need for specialized programme to help them cope with auditory hallucinations so that they can adjust to community living.

### 7.4. Training for Professionals Working with People with Auditory Hallucinations

There are a significant proportion of people with schizophrenia who suffer from auditory hallucinations. However, there has been a general lack of direction in respect of casework, group therapy, self-help groups, family education, and community networks for rendering specific interventions for people with auditory hallucinations. Psychiatric recovery in Hong Kong generally stress medical care, and there is no systematic individual and self-help group service for the voice hearers. As basic mental health training does not cover work with psychiatric patients in depth, not to mention those with auditory hallucinations, there is a need to enhance the training of mental health professionals so that they are equipped with knowledge to help hearers cope with auditory hallucinations. With the implementation of community care policy, professionals, such as psychologists, psychiatric nurses, and psychiatric social workers at different settings have encountered more clients living in the community who face mental health problems, in particular auditory hallucinations. Continuing education programmes for effective practice or intervention with people with auditory hallucinations should be provided so that professionals can bring more knowledge and skills to bear in psychiatric assessment of auditory hallucinations, in particular with regard to their propensity toward self-harm or violence, and in developing interventions with the clients. 

### 7.5. Family Education for Family Caregivers of Voice Hearers

For many Chinese patients with schizophrenia, family members are still their primary caregivers. As a result of the illness, voice hearers may not trust their families enough to share their voices with them. Many family caregivers suffer great emotional strain as they have to tolerate delusions, hallucinations, and accusations from the patients. Many are frustrated with the loneliness and helplessness involved in providing care to their sick family members, as well as in encountering discriminatory attitudes from the community at large. Family education is therefore needed so that the family caregivers may be equipped with knowledge about the prognosis of schizophrenia and become better able to cope with the impacts of the problem of auditory hallucinations. With such knowledge and skills, family caregivers can play a key role in the recovery of the voice hearers. This is an exploratory study on auditory hallucinations of people in Hong Kong; its results suggest that comprehensive research to study the needs of family caregivers of people with schizophrenia in Hong Kong is warranted. 

## Figures and Tables

**Table 1:** Profile of research participants.

Sociodemographic characteristics	No. of participants
Sex	
Male	12
Female	8
Age	
20–29	1
30–39	8
40–49	10
50–55	1
Education	
Primary	8
Secondary	10
Postsecondary	2
Employment	
Sheltered workshop	5
Day activity centre	5
Unemployed	10
Living situation	
Living with family	14
Living with spouse	2
Half-way house	4

**Table 2:** Coping strategies adopted by the participants.

Strategies	Participants
Ignoring the voices	Alfred, Bobo, Catherine, David, Fred, Henry, Gilbert, Ada, Betty, Ben
Asking the voices to go away	Alfred, Carol, Daisy, Joe
Scolding or arguing with the voices	Bobo, Catherine, David, Ivan, Eva, Carol, Ben
Setting boundaries for the voices	Alfred, Catherine, David, Fred, Flora, Edmond, Ken, Joe
Distracting the voices through engaging in other activities, such as listening to music, singing, and reading	Alfred, Bobo, Catherine, Fred, Gilbert, Ivan, Daisy, Flora, Edmond
Jotting down the content of the voices	Alfred, Ken, Joe
Relaxing	Alfred, Gilbert, Jason
Selectively listening to the voices	Alfred, Catherine, Joe
Sharing the voices with family members/friends/professionals	Henry, Gilbert, Ken
Accepting the existence of the voices	Bobo, Joe
Others: decreasing meat intake	Jason, Edmond
